# A Novel Method for Identification and Quantification of Consistently Differentially Methylated Regions

**DOI:** 10.1371/journal.pone.0097513

**Published:** 2014-05-12

**Authors:** Ching-Lin Hsiao, Ai-Ru Hsieh, Ie-Bin Lian, Ying-Chao Lin, Hui-Min Wang, Cathy S. J. Fann

**Affiliations:** 1 Institute of Biomedical Sciences, Academia Sinica, Taipei, Taiwan; 2 Department of Mathematics, National Changhua University of Education, Changhua, Taiwan; The George Washington University, United States of America

## Abstract

Advances in biotechnology have resulted in large-scale studies of DNA methylation. A differentially methylated region (DMR) is a genomic region with multiple adjacent CpG sites that exhibit different methylation statuses among multiple samples. Many so-called “supervised” methods have been established to identify DMRs between two or more comparison groups. Methods for the identification of DMRs without reference to phenotypic information are, however, less well studied. An alternative “unsupervised” approach was proposed, in which DMRs in studied samples were identified with consideration of nature dependence structure of methylation measurements between neighboring probes from tiling arrays. Through simulation study, we investigated effects of dependencies between neighboring probes on determining DMRs where a lot of spurious signals would be produced if the methylation data were analyzed independently of the probe. In contrast, our newly proposed method could successfully correct for this effect with a well-controlled false positive rate and a comparable sensitivity. By applying to two real datasets, we demonstrated that our method could provide a global picture of methylation variation in studied samples. R source codes to implement the proposed method were freely available at http://www.csjfann.ibms.sinica.edu.tw/eag/programlist/ICDMR/ICDMR.html.

## Introduction

DNA methylation, one of the most important epigenetic factors, has been intensively investigated, and its influence in a variety of human diseases, most notably cancer, has been firmly established [1], [2]. In contrast to sequence variation, DNA methylation of cytosine residues at the C5 position has an effect on gene regulation without changing the DNA sequence [3], and this mechanism may therefore make a significant contribution to the missing heritability of complex traits [4]. Advances in biotechnology have led investigators to undertake methylation studies on a genome-wide or whole-genome scale using array- or sequencing-based technologies [5], [6]. Extensively profiling methylation variation either in or between populations—such as in case-control studies or in different tissue types— is crucial for furthering our understanding of the role of DNA methylation in pathogenesis and carcinogenesis.

Variations in DNA methylation exist at various DNA sites, including differential methylation at a CpG site, allele-specific methylation, and haplotype-specific methylation [1]. A differentially methylated region (DMR) refers to a genomic region with multiple adjacent CpG sites that exhibit different methylation statuses among multiple samples and provides the most well-analyzed example of methylation variation. The objectives of DMR studies can be broadly divided into two types: (i) identification of DMRs across populations and (ii) identification of DMRs within a population. Many studies have been undertaken of the former type, in which differences in methylation levels have been explored in individuals with different phenotypic labels, such as diseased and healthy tissues. In such cases, the traditional Student's t-test and Wilcoxon Rank Sum Test (WRST) [7], [8] can be used to find DMRs, using normalized methylation levels between two groups; this has been done using the conventional univariate test for differential expression analysis. In addition, an analysis of variance (ANOVA) model, relying on raw intensity data, has been developed to identify aberrant methylation patterns for oligodendroglioma and breast cancer samples, respectively [9], and “sliding window” approaches, in which various window sizes are used, have also been proposed for methylation segment analyses [10].

The isolation of DMRs across samples in the same population has attracted much recent attention, and biologically distinct subtypes of a disease that may cause molecular or clinicopathological heterogeneity have been recognized as a result [11]. Indeed, DMRs associated not only with different tissue types [12] but also with different disease subtypes, including breast cancer [13], large B-cell lymphoma [14], and acute myeloid leukemia [15], have been identified. Thus, success in the identification of DMRs in a type of cancer may help to discover possible subtypes and could provide new insights into disease progression, which could be used to identify specific drug targets and pharmacogenomics biomarkers [16]. An alternative unsupervised approach can be used to identify DMRs without reference to phenotypic information and can assist investigators in determining methylation variation in the studied samples.

The feature selection method that filters CpGs based on their variability, where features with higher variances are thought to be differential in samples and more likely to be DMRs [17]. Unfortunately, this method lacks the statistic test for determining the significance. A quantitative approach for DMR identification and characterization (QDMR) has been proposed recently using Shannon entropy which measures variation or change in a series of events and has been applied to the study of differential expression genes [18]. In the QDMR, a pointwise method is used in which a weighted entropy score is calculated for each probe to represent the extent of methylation differences across multiple samples. Unlike ranking-based feature selection method, the QDMR provides a statistic for each probe to test the divergence of methylation levels with respect to average methylation level across samples. The methylation statuses of neighboring CpG sites are not independent of each another [7], and it is possible to have positive correlations of methylation intensities in nearby probes across the genome, especially in tiling arrays or from data using sequencing technologies that generate dense data in a specific region of the genome [19], [20]. Aggregating information from neighboring probes, however, cannot be taken into account using the pointwise approach, although appropriately incorporating this information into the analysis of DMRs may reduce false positives, because the methylated fragments are always longer than the probe length used in the array. Here, Identification of Consistently Differentially Methylated Regions (ICDMR), an unsupervised approach, is proposed to directly analyze methylation intensity data generated from tiling arrays to locate DMRs across a large set of samples simultaneously. This method considers all correlations of signals between nearby probes, i.e., those that are biologically significant and those that are not. The former correlation arises from changes in DNA methylation status, whereas the latter arises from the intrinsic correlation of probes, such as the linear correlation arising from overlapping probes or from the hybridized DNA fragments spanning multiple probes on the array [21], [22]. The proposed method provides a way to calculate the concordance between adjacent probes, where concordance measures the consistency of methylation status between two probes among individuals. A population-based distribution is also used to assess the significance of the concordance. Thus, contiguous probes with significant concordance can then be integrated to form a consistently DMR. In other words, the proposed method searches for the region(s) showing different methylation statuses among individuals in a population, where these differences are consistent across the probes in the region.

## Methods

Two different measurements, M and β, are frequently used to assess the methylation level [23]. The β, varying between 0 and 1, is reported as a ratio of methylated intensity to the sum of methylated and unmethylated intensities. Although β provides an absolute measure of DNA methylation level and is easily interpreted, it imposes serious challenges when applying to many statistic models with a heteroscedasticity in the low and high methylation levels [24]. The M, ranging over all real numbers, is calculated as a log-ratio of the methylated and unmethylated intensities. The M value is more statistically valid to common statistical tests used in gene expression study, and has been suggested to be related with β by a log2 logistic transformation [17],
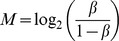



### CpG island methylation data from human astrocytomas

The dataset of CpG island hypermethylation in human astrocytomas [25] was obtained from the National Center for Biotechnology Information's Gene Expression Omnibus (GEO), accession number GSE19391. It consisted of six normal tissues and 30 astrocytomas. The Human DNA Methylation 385 K Promoter Plus CpG Island Array (Roche NimbleGen, Madison, WI) had been used for assaying methylation levels in 36 samples for 28,000 CpG islands and 18,000 promoters, using nearly 385,000 probes spread over the genome. The astrocytoma data included samples belonging to all four grades of the World Health Organization's tumor classification system: 6 grade I (or T1) samples, 7 grade II (or T2) samples, 9 grade III (or T3) samples, and 8 grade IV (or T4) samples. The normalized log_2_ ratio data were used directly, and only the autosomal probes were considered in the analysis of the DMRs

### DNA methylation data from human tissues

The DNA methylation data from human liver, frontal cortex, spleen and colon [26] were obtained from GEO (accession number GSE23841). The dataset was generated by genome tiling array, using genomic DNA hybridized to custom-designed NimbleGen microarrays (CHARM human array v 1), and consisted of five samples each of normal liver, frontal cortex, spleen, and colon and five samples of colon cancer tumors. These data were first used to identify a large number of tissue-specific DMRs and cancer-specific hypermethylation and hypomethylation in CpG island shores in colon cancer tumors using a supervised approach. Because the normalized data represent the fraction of methylation (represented as a decimal value ranging from 0 to 1), the logistic link function was applied to convert the data into the form of a log_2_ ratio [17]. These transformed data were then used to examine the ability of ICDMR to identify DMRs across different human organs, and/or between health and diseased tissue.

### ICDMR: Clustering methylation data by a normal mixture model

In order to better estimate methylation status in studied samples, we propose to exploit the bimodal distribution of M. For a probe, say *d*, we first estimate the probabilities of each individual's methylation status by using relative methylation intensities across samples. Consider *n* methylation intensities, observed for a probe, *d*, denoted as *m_d_* = (*m_d_*_,1_, *m_d_*_,2_,…, *m_d_*_,*j*_,…, *m_d_*_,*n*_)*^T^*, where *m_d_*_,*j*_ is the log_2_ ratio of the intensities of treated versus untreated DNA for the *j*th sample. In gene expression studies, the model-based clustering method is a frequently used technique to identify groups of cohesive observations, and it assumes that the data arise from a normal mixture model [27]. In contrast to gene expression data, methylation data follow a bimodal distribution, corresponding to methylated and unmethylated regions [28]. In view of this, we propose a separation of methylation intensities, *m_d_*, if methylation statuses are different between two groups of individuals. A traditional normal mixture model with the number of components fixed at two was therefore implemented to describe the pattern of methylation intensity in the samples. Under a univariate normal mixture model, with component number equal to two, the likelihood, *L_d_*, of observing the methylation intensities, *m_d_*, can be expressed as
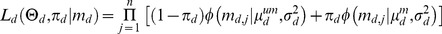
where 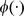 and 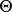 are the density function and parameters of the normal distributions, and 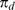 is the prior probability of samples arising from the methylation group. The parameters 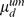 and 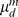 are the mean intensities for unmethylated and methylated groups, respectively, and 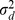 is the common variance between groups. This mixture model can easily be fitted by the expectation-maximization algorithm, resulting in maximum likelihood estimates, 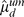, 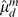, 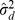 and 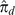, of the model parameters [29]. Thus, using Bayes theorem, the estimated posterior probability, *p_d,j_*, that the *j*th individual is methylated is given by
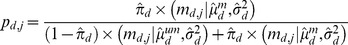



This process is carried out for each probe in the array to produce a posterior probability matrix, 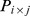, where the rows *i* = 1,…,*d*,…,*t* are sorted according to their physical positions on the chromosome. The value of *t* is the total number of probes in the array. The posterior probabilities are then utilized as the estimated methylation status to quantify concordance of methylation status in samples between probes.

### ICDMR: Scoring concordance between probes in clustered samples

To quantify the similarity of posterior probabilities between two neighboring probes, a simple score of concordance, *c_d_*, is utilized as
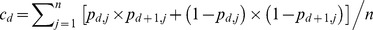
where *d* = 1,…, (*t* − 1). The *c_d_* ranges from 0 to 1; a larger value represents higher concordance and is more likely to arise from probes located at a DMR. The *c_d_* reaches a maximum of 1 if for each individual, *j,* the following conditions are met: (i) the estimated probabilities of methylation, *p_d,j_* and *p*_(*d*+1),*j*_, are either 1 or 0 and (ii) the estimations are the same between adjacent probes, i.e., *p_d,j_* = *p*_(*d*+1),*j*_. In other words, the methylation statuses across samples are consistent between probe *d* and (*d*+1), and the posterior probability for each individual is equal to 1 for either the unmethylated or methylated state. If the methylation intensities are similar across samples, i.e., the region is not a DMR, the posterior probabilities would be near 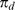 for most of the samples. In a fair-coin-tossing setup for a non-DMR probe, the distribution of *c_d_* would be symmetric and centered on the value 0.5. Hence, the concordance could be used to represent the degree of agreement in separating methylated and unmethylated individuals between probes.

### ICDMR: Determining the threshold for DMRs

When the concordances are observed for all probes in the array, an objective threshold is required to determine which region on the chromosome exhibits a cluster of unusually high concordant scores, i.e., which region is a DMR. The distribution of the scores is a mixture of DMRs and non-DMRs, and the proportions of the components are difficult to estimate without any prior knowledge, such as the distribution of *c_d_* for DMRs. One of the practicable approaches in studies of Chip-enriched region is adopted in the present study [30]. In brief, the *c_d_* originated from the probes resided in DMR is stochastically larger than that from non-DMR and the mode of *c_d_* calculated from non-DMR would be nearing 0.5 under a fair-coin-tossing setup. Thus, scores <0.5 were used to estimate the distribution of non-DMRs, by mirroring its distribution over 0.5 to generate a symmetrical distribution, ranging from 0 to 1 with a mode at 0.5. This strategy neglects possibility of extension of alternative distribution to the left of 0.5 and might result in more conservative results. Whereas the null distribution was estimated, the DMR threshold was directly computed using the sample percentile. For instance, given a type I error rate equal to α, the threshold, *T*_α_, will be the (1 − α)th percentile of the estimated null distribution, and contiguous probes with concordance scores larger than *T*_α_ will then be aggregated to form a consistently DMR.

### ICDMR: Correction of non–biologically relevant correlations between probes

It has been demonstrated that methylation data observed from array based methylation platforms display a positive nature dependence structure between neighboring probes [30]. This dependency is a spatial correlation among nearby CpG loci and could be due to experiment factors such as probe affinity, PCR amplification and DNA fragment size [31], etc. Previously, the nature dependencies among neighboring probes were proposed by an autocorrelation model [30], [32]. In a study of DMR with multiple samples, the correlation of methylation intensity between neighboring probes is contributed both from the spatial correlation and methylation status within samples. Therefore, in this study, the methylation intensity correlation was decomposed into two parts, namely continuous and discrete, the former referred to the nature correlation inherent in the experiment, and the latter was for the correlation due to changes of methylation status in samples between probes (Figure S1).

In a methylation study with *n* samples, the methylation intensities of a sample *j*, *m_i_*_,*j*_ for *i* = 1,…,*t*, vary greatly between neighboring probes and intuitively the average methylation intensity of a probe *d*,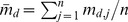, could be sensitive to the sample ratio between methylated and unmethylated groups. In the normal mixture model, both proportions and distributions of methylation intensities for methylated and unmethylated groups are estimated. Therefore, for a pair of neighboring probes, *d* and (*d*+1), the weighted averages, 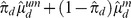 and 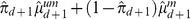, of methylation intensities estimated from the mixture model are subtracted from the methylation intensities, *m_d_* and *m_d_*_+1_, respectively, to obtain the centralized intensities 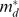 and 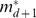. The process of weighted mean shift is a way to normalize methylation intensities between probes, and takes the difference in the proportion of samples being methylated between probes into account. Such normalization has no effect on the clustering results or the posterior probability matrix, *P*.

To remove only the continuous correlation, a weighted least square regression model is performed
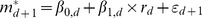
with weight, *w_d_*, where 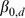 and 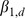 are the coefficients of the regression model and ε is the error term. The residuals, 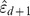, where 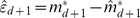, are calculated to represent the first-order correction of methylation intensity, 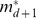. A value of *p_d_* is used for the weight, (*w_d_* = *p_d_*), if 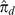 is >0.5; otherwise, a value of (1−*p_d_*) is used. The independent variable *r_d_,* where *r_d_* = (*r_d_*_,1_,*r_d_*_,2_,…,*r_d_*_,*n*_)*^T^*, gives the methylation intensity of 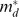 after adjusting for the variations caused by the different methylation status of probe *d,* where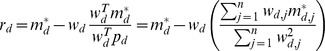.

The adjustment constant calculated in the parenthesis (on the right side of the equation) is equal to the coefficient estimated in a linear model, by regressing *w_d_* on 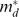 without including an intercept term. Such estimation takes the uncertainty of clustering into account by using posterior probability when methylation intensities are not segregated with certainty to unmethylated and methylated samples. In case of complete separation, e.g., all elements in *p_d_* are equal to 0 or 1, the adjustment constant will be the arithmetic average of the methylation intensity of methylated groups. Pretreatment of the methylation intensities of probe *d* by consideration of the potential variation of methylation in the samples avoids any discrete correlation between probe *d* and *d*+1, thereby diminishing its effect on the model by removing this from the continuous correlation component. The corrected methylation intensity, 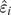, for *i* = 2,…*d*,…*t*, is then used to recalculate the mixture model and compute the corrected posterior probability, 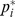 and score of concordance, 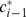.

### Simulations

To justify the efficiency of this method, several log_2_ intensity ratio data matrices, consisting of 50 rows (samples) and 30,000 columns (probes), were simulated using the autoregressive model used by Kuan *et al.* [21], by considering only the first-order correlation in the present study, i.e., AR(1),
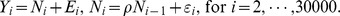



*N_i_* is the autoregressive background. The result of the tiling array experiment is a series of intensity measurements along the genome and these measurements are positively correlated [21], [22]. In this study, to take into account this nature dependency, the value of ρ was set at 0, 0.3, 0.5 and 0.7, to represent zero, low, moderate, and high correlation of background intensity measurements among neighboring probes, respectively. In order to compare with supervised methods, the 50 samples were partitioned into 25 cases and 25 controls, and the change of methylation status was randomly assigned to cases only, i.e. 25 controls were all unmethylated. *E* was the real signal for methylation intensity and it determined differences of intensities between methylated and unmethylated groups. The distribution of M was studied previously by Du, et al., [24], and results showed that M ranged within (−∞, ∞) with one negative mode (unmethylated mode) and one positive mode (methylated mode) located within (−∞, −2) and (2, ∞), respectively. Accordingly, we used *E* = 0 for unmethylated group and *E* = 2 and 4 for methylated group in this study. The size of a DMR was fixed at 10 probes, and start sites for the region were arranged randomly to satisfy scenarios where the proportion of probes residing in the DMRs equaled 0.05 or 0.2 for 30,000 probes [21]. The methylation frequency (MF) was the proportion of methylated cases in the entire case samples as defined previously [33], [34]. In our study, the effect of MF on performance (sensitivity, specificity) was studied by considering MFs of 0.1, 0.2, 0.4, 0.6, 0.8 and 1, respectively. For example, MF = 0.2 meant 20% (5 out of 25 cases) were simulated from the AR(1) model with *E* = 2 (methylated group) and 80% (20 out of 25 cases) were from *E* = 0 (unmethylated group). In reality, MF
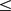
0.4 was often found in previous methylation studies [34], [35].

When the simulation data consisted of both DMRs and non-DMRs, sensitivity and specificity were estimated as the performance for identifying DMRs and non-DMRs, respectively. For each simulation, 30,000 observed test statistics were analyzed independently for each of the QDMR, t-test and WRST, where 29,999 observed concordance scores were used for ICDMR. For each method, the false positive rate (FPR) expressed as 1
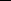
specificity was calculated as the probability that the test statistics observed from probes resided in non-DMRs been detected with statistical significance. The sensitivity was calculated as the probability that the test statistics observed from probes resided in DMRs, also identified with statistical significance.

## Results

### Distribution of concordance of probes found in non-DMRs

The simulation study was first carried out under the null hypothesis, i.e., there is no DMR among the samples, using data generated from the AR(1) model and with ρ values set at 0, 0.3, 0.5, and 0.7. Ten repeats were performed, and 30,000 probes with 29,999 concordance scores were produced in each simulation for each value of ρ. The expected trend that score of concordance approached 1 with increasing correlation between probes was found for the raw data (without correction for the intrinsic correlation between probes), but the bias was largely eliminated after implementing the correction procedure. After correction, the distributions were all nearly symmetrical around a concordance value of 0.5, although a slight shift of mode is apparent for the high correlation values (Figure S2A). The variation of concordance between different correlations prevented determination of the significant threshold for the raw data, e.g., thresholds of uncorrelated probes (ρ = 0) differed greatly from those of correlated probes (ρ = 0.3, 0.5, and 0.7). In contrast, thresholds were in the range of 0.725 to 0.735 after correction, giving more consistent values across repeats or among different correlation structures (Figure S2B). In other words, the observed properties of concordances estimated from the corrected data, i.e., their approximate symmetry and independence of the correlation structure, make it possible to find a universal threshold for determining DMRs in a whole-genome study. Thus, only the method that incorporated the correction of correlations was considered for subsequent analyses.

### Efficiency of ICDMR in distinguishing between DMRs and non-DMRs

**Figure 1:**
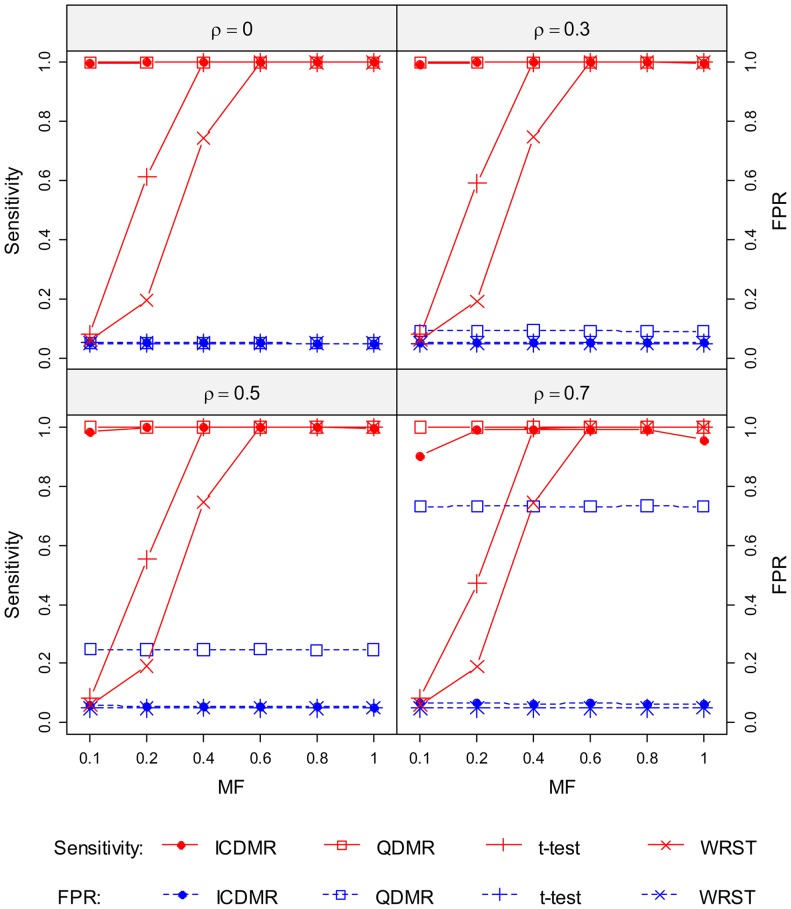
Sensitivity and false positive rate. The figure summarizes mean sensitivity (red solid line, left axis) and false positive rate (blue dash line, right axis) for ICDMR, QDMR, t-test and WRST. The mean difference of methylation intensities between methylated and unmethylated groups is 2 (i.e., *E* = 2). The proportion of probes residing in the DMRs is 0.2. At the indicated MF, mean sensitivity and false positive rate are calculated given the correlation between neighboring probes being 0 (ρ = 0), 0.3 (ρ = 0.3), 0.5 (ρ = 0.5) and 0.7 (ρ = 0.7), respectively. Different MF values are indicated on the x-axis.

Some of the commonly used supervised methods in studying DMRs between two comparison groups were t-test and WRST [7], [8]. To compare performances of supervised and unsupervised methods for determining DMRs and non-DMRs, the sensitivity and FPR were calculated given the level of significance α = 0.05. All of the four comparative methods showed satisfactory results for preserving a FPR of approximately α = 0.05 when methylation intensities were independent across probes (Figure 1, ρ = 0). With the same FPR, the sensitivities were lower for the two supervised methods when MF
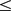
0.4 whereas the sensitivities were similar to those of the two unsupervised methods.

In the dependent scenarios, i.e. ρ
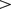
0, the supervised methods showed similar results comparing with those from independent scenario (Figure 1). Given the same ρ, the results showed that MF had a strong and positive impact on the performance of supervised methods in identifying DMRs. For unsupervised methods, the sensitivities of QDMR were almost one regardless of the value of ρ, however, the FPR increased dramatically with higher values of ρ. For example, for QDMR, the FPRs were 0.09, 0.25, and 0.73 for low (ρ = 0.3), moderate (ρ = 0.5), and high (ρ = 0.7) correlations, where, for ICDMR, they were 0.052, 0.055 and 0.063, respectively (Figure 1; Table S1). The sensitivities of ICDMR were similar to those of QDMR across all scenarios, except for ρ = 0.7 and MF = 0.1 (90.3%) or MF = 1 (95.6%).

With the level of significance α = 0.05, these results suggested that, for supervised methods, the performances of identifying non-DMRs were always better than those of unsupervised methods, but the performances of identifying DMRs declined when MF values were smaller (MF<0.4) regardless of the value of ρ. For the unsupervised methods, the performance of QDMR to identify DMRs was high even when methylation aberration was only present in minor cases, however, performance for identifying non-DMRs dropped when the correlations between neighboring probes increased. In contrast, for ICDMR, the performances of determining DMRs and non-DMRs were more robust across different values of ρ and MF (Table S1).

When the methylation intensity difference between methylated and unmethylated samples became larger as *E* increased from 2 to 4, higher value of *E* had no influence on the FPRs of the supervised methods. When MF was low, the sensitivities of supervised methods were always lower than those obtained from unsupervised methods regardless of the *E* value (Figure S3). For unsupervised methods, the FPRs of QDMR were high (>0.7 given ρ = 0.7) regardless of the *E* value, while the FPRs of ICDMR remained low (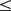 0.065 given ρ = 0.7). The results showed that the sensitivities of QDMR were close to 1 regardless of the *E* value. For ICDMR, the increases in sensitivities were larger as *E* increased given ρ = 0.7, e.g. the sensitivity ranged in (0.90, 0.99) and (0.99, 1.00) for *E* = 2 and 4 given ρ = 0.7, respectively. These results suggested that, when ρ = 0.7, the slightly lower sensitivity observed from ICDMR may be due to that the discrete correlation might had been mistaken as continuous, and the difference in methylation intensities between methylated and unmethylated groups was then removed in the process of correlation correction as a result of the expansion of overlap between methylated and unmethylated samples.

In this simulation study, we also assessed whether the proportion of differentially methylated probes affected performance. Results observed for a proportion of 0.20 were in agreement with those for a proportion of 0.05 (data not shown). This finding suggested that the distributions of sensitivity and FPR were independent of the proportion of DMR in the data.

### Human astrocytomas

To identify potential DMRs, a dataset comprising methylation profiles of normal and cancerous cells, each of which covered 32,239 CpG islands and RefSeq promoter regions included at least two probes per island and region, was analyzed. The analysis showed 336,963 concordance scores, spread over the autosomes; ∼16% of these (54,257) had significant threshold values of 0.786 or greater (α = 0.05; Figure S4). Significant concordances were found in 31,015 non-overlapping DMRs. Of these DMRs, >60% had only two probes. To reduce the false-positive rate of DMR detection, only regions with at least three contiguously significant concordances were pursued. Thus 5,208 DMRs, located in 4,684 CpG islands, were identified, and these ranged in length from 275 to 20,000 bps (base-pairs), with a mean length of 440 bps.

For each DMR, the mean frequency of samples belonging to a methylated group and the mean posterior probability of each individual belonging to a methylated group were estimated in the mixture model and calculated across probes residing in the consistently DMRs. The vast majority of DMRs were identified by some specific and high methylation intensities in minor samples, e.g., the distribution of the mean frequency was shifted toward <0.5, whereas most proportions were <0.2 (Figure S5).

**Figure 2:**
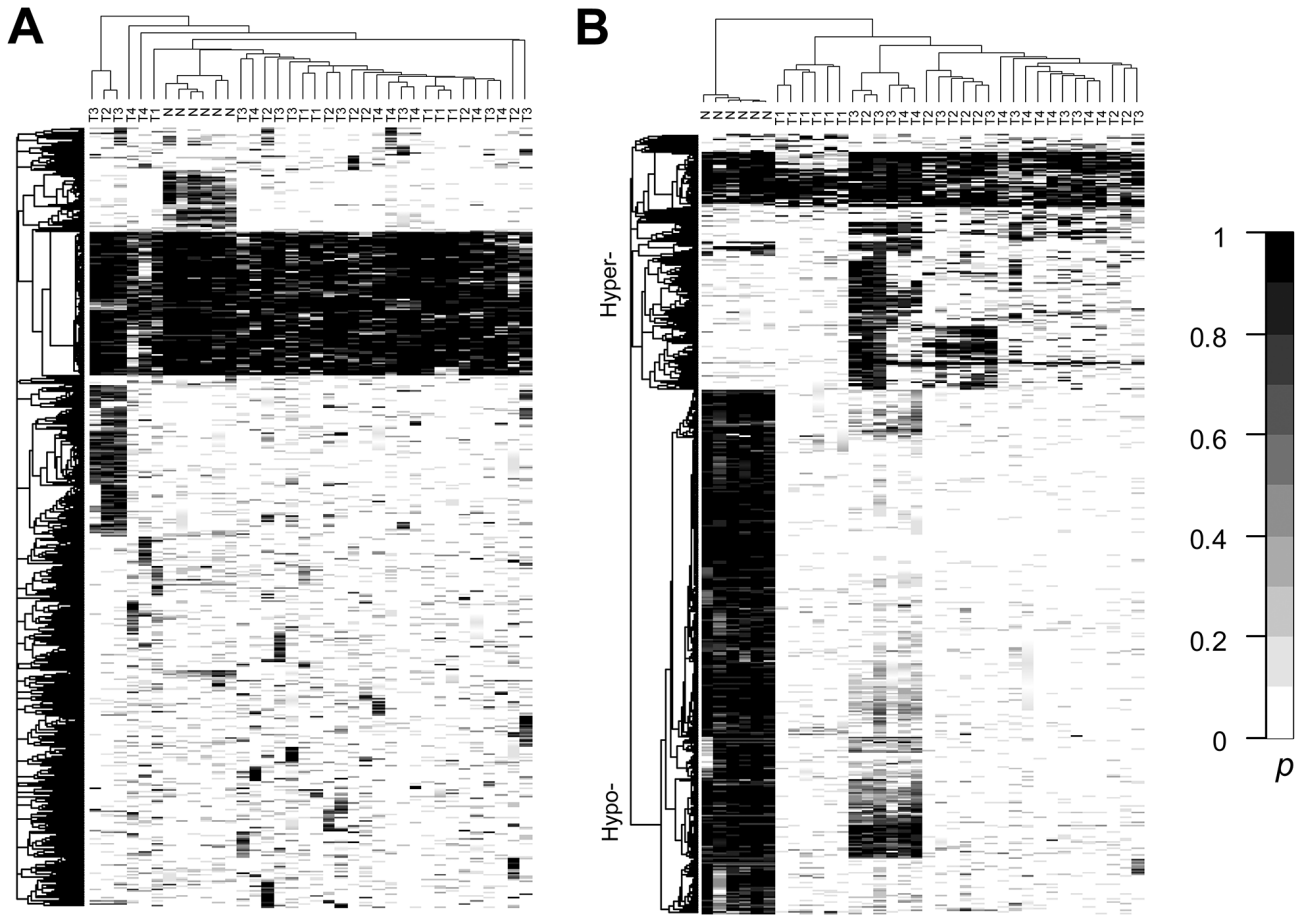
Heatmap of posterior probabilities for human astrocytoma samples. Hierarchical clustering of the posterior probabilities is displayed as a heatmap for (**A**) DMRs with a low degree of differentiation (2,652 DMRs) and (**B**) DMRs with a high degree of differentiation (2,556 DMRs) in 30 tumor (T) and 6 normal (N) samples. The intensity is proportional to the degree of methylation, as indicated in the figure.

To visualize the correlation between DMRs and samples, DMRs were divided into two groups: those with the mean frequency of methylation lying inside the range of 0.15–0.85, and those lying outside of this range (Figure S5). There were 2,556 DMRs (49%) and 2,652 DMRs (51%) in each respective group, corresponding to DMRs with a high and low degree of differentiation, respectively. The clustering patterns of the posterior probabilities across the samples showed that DMRs with a low degree of differentiation were largely due to hypermethylation in a small number of cancerous samples that had disorderly methylation profiles within their DMRs (Figure 2A). For sample clustering, only the six normal cell samples were organized as a distinct group. In contrast, clustering patterns of DMRs with a higher degree of differentiation were more clearly visible either across samples or DMRs (Figure 2B).

The methylation status of the normal cells was relatively consistent as compared with that of the tumors. All normal cells were tightly clustered together and were distinct from cancerous cells. In addition, grade 1 tumors (T1) were separated completely from other higher-grade tumors, as can be seen in the vertically clustered tree shown in Figure 2B. In addition, three major clusters were formed by tumors having a tumor classification of >1: a mixture of one T2, three T3, and two T4 tumor types; one T2- and T3-specific cluster with four T2 and two T3 tumor types; and the largest group, which consisted of two T2, four T3, and six T4 tumor types. For the DMRs, two major groups were observed for tumor hypermethylation (836 out of 2,556 DMRs) and hypomethylation (1,720 out of 2,556 DMRs) and are respectively indicated by the upper and lower branches visible in the horizontal cluster tree shown in Figure 2B.

To localize tumor hyper- and hypomethylation regions within the genome that are related to specific genes, the distance from each DMR to its nearest gene was calculated using the software ChIPpeakAnno, an R package [36]. 43.3% (362 of 836) of hypermethylation regions and 19.2% (331 of 1720) of hypomethylation regions fell within 1,000 bp upstream of transcription start sites for 350 and 304 unique genes, respectively. Because changes in methylation status within this putative promoter region are crucial for regulating gene expression [37], we performed functional analyses of these genes using the Functional Annotation Clustering Tool, part of the Database for Annotation, Visualization and Integrated Discovery (DAVID) software suite [38]. We found that the 350 genes that were close to hypermethylation regions were mostly related to transcription regulation, embryonic morphogenesis, and neuronal fate commitment as the top three enriched clusters (Table S2). The top two clusters for genes identified as being close to hypomethylation regions were a cluster of genes involved in spermatogenesis and a group of eleven genes with ankyrin repeats, which are one of the most common protein-protein interaction motifs [39].

### Human tissues

DNA methylation profiles vary across human tissues from the same individual, as well as between individuals, and this is known as tissue-specific methylation [7], [40]. The dataset generated by the CHARM arrays contained more than two million probes located in 20,588 autosomal regions [41]. ICDMR analysis of the data, including five distinct tissue types, showed that >230,000 concordance scores (∼11% of the 2,084,540 scores) were larger than the threshold value of 0.785, as determined at α = 0.05 (Figure S6). Using the same criteria that only regions with at least three contiguously significant concordances be considered, 17,601 DMRs, with mean fragment length of 240 bp, were found in 9,038 unique regions.

**Figure 3:**
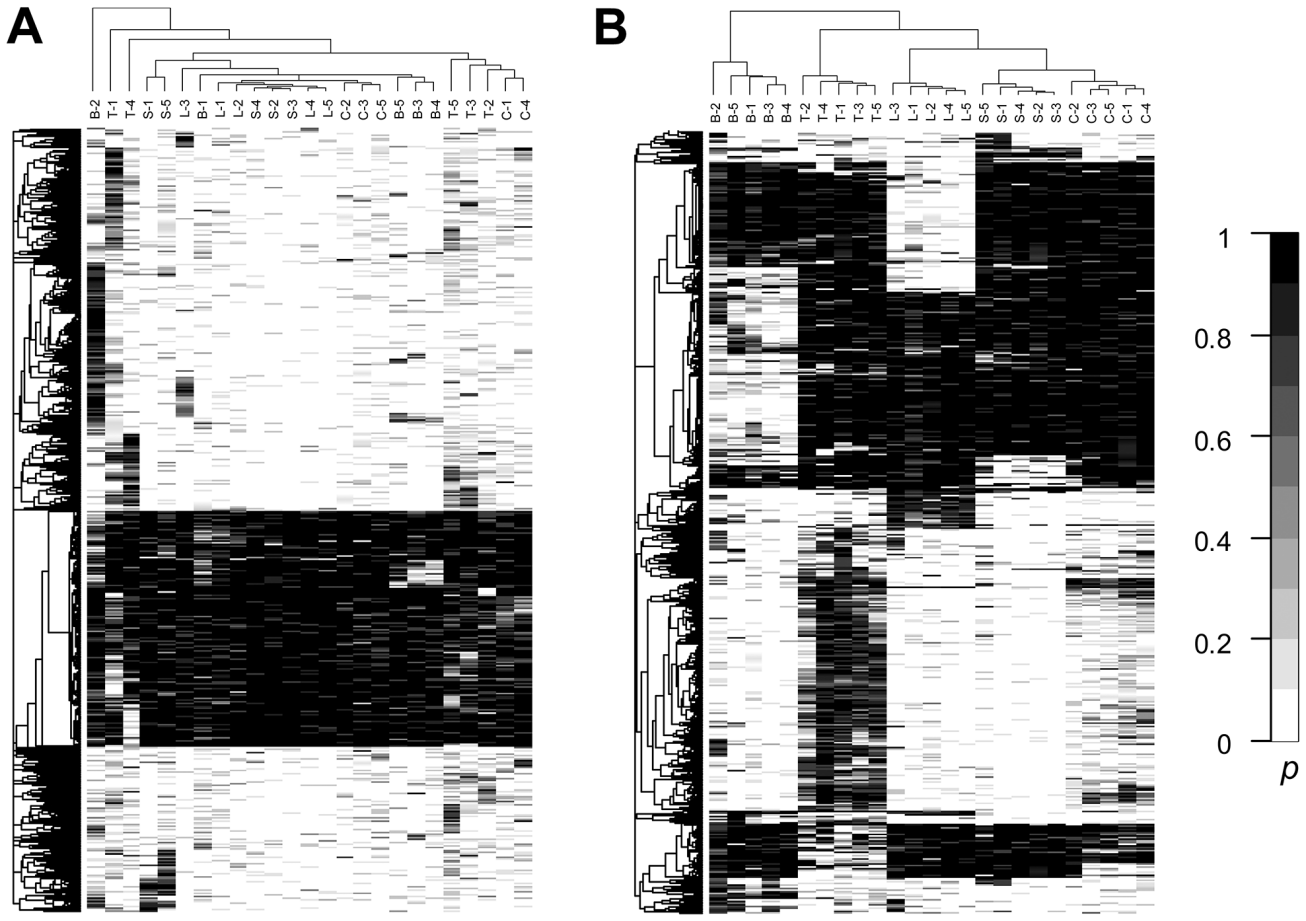
Heatmap of posterior probabilities for various human tissue samples. Hierarchical clustering of the posterior probabilities is displayed for (**A**) DMRs with a low degree of differentiation (6,051 DMRs) and (**B**) DMRs with a high degree of differentiation (1,550 DMRs) in 5 liver (L), 5 frontal cortex (B), 5 spleen (S), 5 colon (C) and 5 colon cancer (T) samples.

To further investigate how different tissue types can be classified according to methylation profiles, these 17,601 DMRs were divided into two groups according to their degree of methylation variation, as described previously. Thus, 11,550 DMRs had a low degree of methylation variation and 6,051 DMRs had a high degree of methylation variation. Clustering results indicated that most of the DMRs with a low variation in methylation arose from diversity between individuals (interindividual), e.g., there was no tissue type that could be grouped divergently from all other tissues (Figure 3A). For DMRs with higher methylation variability, five distinct sample clusters emerged on the hierarchical structure corresponding to five different tissue types, as shown in the vertical tree in Figure 3B. For four different normal tissues, the clustering branches indicated that the methylation profile for colon tissue was closest to that of spleen tissue, followed by liver and then brain; this reflects the similarity of biological functions among these organs. Interestingly, this clustering pattern is similar to that obtained from a gene expression study by Son *et al.* [42], where 19 different organs from 30 different individuals were analyzed.

Methylation of promoter CpG islands is associated with silencing of gene expression [43]. A tissue-specific gene expression study revealed that liver and brain have a relatively large number of specifically expressed genes, in contrast to colon and spleen [42]. This is consistent with our results, where DMRs were considerably less methylated in liver and brain, as compared with their levels of methylation in the colon and spleen (Figure 3B). In addition, many hypermethylation regions were detected for the colon tumor, whereas most of these regions were methylation free in the other normal tissue samples. These 6,051 high-variability DMRs were mapped to the transcription start site of 1,051 unique genes, and the Benjamini-Hochberg False Discovery Rate multiple testing correction was used to calculate the adjusted *p*-values for these. Genes involved in developmental processes were found to be over-represented in the annotated cluster (*P*-value = 1.1×10^−13^), including genes for the homeobox sequence (*P*-value = 2.1×10^−12^), for neuron differentiation (*P*-value = 6.0×10^−8^), and for embryonic organ development (*P*-value = 2.1×10^−5^; Table S3).

## Discussion

DMRs, one of the most important methylation variants in populations, have been described in various contexts, including imprinting-specific, tissue-specific, reprogramming-specific, cancer-specific and aging-specific functions [1]. The differences between these contexts depend on what types of samples were collected and which phenotypes were determined in advance of the comparisons. Recent studies have suggested that many DMRs associated with diseases only show methylation aberration in a smaller portion of cases, always in less than 40% of cases [33]–[35]. We adopted such concept and carried out a comprehensive comparison. Our results showed that unsupervised methods always outperform supervised methods in identifying DMRs.

In this study, we proposed a novel DMR identification method, namely ICDMR, and compared it with a recent method QDMR. With the need of test statistic for comparisons of performance in different methods given a type I error, we therefore chose QDMR as a comparative method in this study. ICDMR is an unsupervised approach, where there is no need to choose the comparative group in advance, and is able to identify methylation modifications with low and high degrees of differentiation in studied samples. It took about 2 hours to analyze 36 samples with data from 385,000 probes and 12 hours for 25 samples with data from 2.1 million probes, respectively, using CPU Intel 3.07GHz and 12 GB main memory running under windows operating system.

M follows a bimodal distribution and seems to be more reliable for detecting DMRs than β, because of their homogeneity of variance between methylated and unmethylated regions [24]. A normal mixture model, comprising two components with a common variance in M between methylation and methylation-free groups, is therefore adopted in ICDMR. On the other hand, β is more widely used than M in experiments of methylation sequencing. We choose to use M over β because of the requirement of normal distribution assumption of methylation intensities across samples in ICDMR. It is possible to replace M by β, if a conversion by using a logistic link function is carried out [17]. In this study, E = 0 and 2 which are equivalent to β = 0.5 and 0.8 are utilized to represent methylation level for unmethylated and methylated groups, respectively. When comparing data generated with E = 0 to the data with E = 2, the scenario describes a case of ICDMR in identifying DMRs with a methylation level difference of 30%. This medium/high methylation patterns occur frequently to the DMRs in imprinted regions or the intergenic regions for some cancers.

Detection of regions displaying concordant methylation profiles in samples between probes is ideally suitable for high-density methylation data, such as data from tiling arrays. With such dense probes where the nature of the dependence structure of methylation measurements between neighboring probes exists [32], our simulation study demonstrated that many spurious signals were observed when the probe data were analyzed independently. In our simulation study, although QDMR yielded a higher sensitivity in general, the false positive rate could reach up to 0.7 when the correlations between probes were high. In cases where difference in methylation signals between methylated and un-methylated groups was small, i.e. *E* = 2, the ICDMR might somewhat lose sensitivity because the edge of signals between methylated and unmethylated groups were hard to define. Therefore, for a methylation study with sparser coverage, where interrogation of a set of known methylated loci is equipped (e.g. Illumina Infinium HumanMethylation27 BeadChip, Illumina, San Diego, CA, USA), the point-wise method (e.g. QDMR) may be more suitable [44]. That is because these probes are always farther apart from each other with low correlation and therefore could be considered as independent [45]. However, for situations of dense probes with high correlation (e.g. CHARM human array, Roche NimbleGen, Madison, WI), ICDMR performs better than QDMR in controlling FPR and retains a comparable sensitivity with QDMR.

The nature of tiling array is that the probes spanning a genomic region are always at a regular interval, that is, the probes are nearly evenly spaced. The median interval length between two spatially consecutive probes are about 50 bps and 37 bps for the arrays used in the study of human astrocytomas and human tissues, respectively. Most of the DMRs reported in the literature ranged from a few hundred to a few thousand bp [23]. Therefore, it is highly likely to find a few probes within a DMR where these probes are correlated. For the methylation profiled with comprehensive genomic coverage by sequencing technology, such as bisulphite-sequencing data, the density of data point is with a base-pair resolution, which is much denser than array based. The extension of ICDMR to deal with such high density data is possible because the method has considered the spatial correlation in its framework. Further study is needed for the extension, since sequencing depth and correlation of methylation levels between nearby CpG sites may affect performance of statistical methods in quantifying methylation levels [46].

A simulation study demonstrates the effects of non–biologically relevant probe signals in determining DMRs, because a lot of spurious signals will be produced if the methylation data are analyzed independently of the probe. With ICDMR, although the estimated FPR is consistent for a given value of α, it is still possible to generate a large number of false positives when analyzing on a genome-wide scale. For methylation studies, a sliding window approach has been frequently used for tiling arrays [19], [47], [48]; the efficiency of detection of this method is clearly dependent on window size, i.e., the sensitivity is higher when the methylation fragment length is perfectly covered by the window size. In the approach described here, ICDMR was used to measure the concordance between adjacent probes, and then contiguously significant probes were grouped to yield consistent and unbiased identification of DMRs. This enabled DMRs of any length to be detected in a single screen and further filtered the resulting DMRs based on the number of probes in the region. For example, from the simulation study, limiting a DMR to a region comprising at least three and at least four probes reduced the estimated FPR to 0.007 and 0.001, respectively, for a given α = 0.05 (data not shown). In other words, an appropriate threshold of extent of contiguously significant concordance in determining DMRs can help to mitigate the effect of false positives in a large-scale study. A serious concern of using the alternative strategy of microarrays is in determining an optimal cut-off point, which needs to be determined for each dataset.

By using QDMR to identify DMRS for the two real datasets, the results have shown that 176,789 (48%) and 1,448,166 (78%) of the studied probes suggested significant low entropy values (*p*-value<0.05) in human astrocytomas and human tissue dataset, respectively. When applying hierarchical clustering among samples in human astrocytomas with 176,789 DMRs, the clustering diagram shows a heterogeneous clustering pattern where no one group is consisted of samples with only one histological grade (Figure S7). For clustering analysis among samples in human tissues with the 1,448,166 DMRs, the clustering pattern is not well correlated in a biologically-relevant manner (Figure S8). It seems that the ambiguous clustering results might be due to a large number of false positive DMRs identified by QDMR as suggested by the results of our simulation study.

For the study of human astrocytomas, the methylation pattern of tumor cells is more variable than for normal brain tissues, for DMRs with either low or high variability. This is consistent with a study of prostate cancer [49] and colon cancer [26], where greater heterogeneity in the methylation profiles is found among tumor samples, as compared with benign adjacent samples, and adds support to the idea that tumors, in general, have highly heterogeneous DNA methylation patterns. Hierarchical clustering showed that the methylation profiles of the DMRs in astrocytomas fell into four distinct groups. This segregation of the analyzed tumor samples was partially correlated to the histological grade, especially for the tumors labeled T1, which are all grouped together. Once a DMR is identified using an unsupervised approach, this could help in discovering cancer subtypes associated with clinical or molecular characteristics, similar to the identification of molecular subtypes by gene expression profiling [50], [51]. This type of methylation signature needs further investigation and has the potential to be adopted for cancer diagnosis, prediction of treatment outcome, and therapy selection [52].

In addition, results from functional analyses indicate that the hypermethylated DMRs of astrocytomas located in the promoter region of genes are highly related to DNA binding factors and transcription; this is particularly true for homeobox genes. These genes are functionally important, and their aberrant methylation may give rise to the modulation of transcription levels for many genes, including those involved in cancer development [53]. Interestingly, in addition to finding hypomethylated DMRs near genes involved in spermatogenesis, we found 12 hypomethylated DMRs near the start sites of 11 other transcripts: the *POTE* gene family of *POTEA, POTEB, POTED, POTEE, POTEG*, and *POTEH* and *ANKRD30A, ASZ1, Fem1a, FANK1*, and *TRPC7*, all of which include a cluster of ankyrin repeats. The expression pattern of *POTE* has been examined in a wide range of human cancers and normal tissue and is considered a member of the cancer-testis antigen class [54]. Recently, hypomethylation of *POTEH* has been proposed as a new epigenetic biomarker for glioma prognosis [55]. However, from an analysis of the methylation signals in the normalized data, we found the vast majority to give a positive result in the DMRs both for normal cells and tumors (Figure S9). Thus, if the decision rule is based on an absolute value of 0, instead of a positive value representing methylation and negative values indicating the absence of methylation, this would lead to most samples being deemed methylated. This may be why the cluster of ankyrin repeats was not found in the tumor-normal DMR group in the original study of astrocytomas [25]. Finding an absolute cut-off point of methylation intensity for identifying methylation across an entire set of probes is quite difficult, because the distribution of methylation signals for a particular probe is subject to the CpG density and amplification [56]. Instead, ICDMR emphasizes the relative methylation signal among samples and is therefore better at correctly identifying DMRs.

In the study of human tissues by using ICDMR, our results have shown to be similar to that obtained from the original study which used a supervised method. Both methods found 5 distinct groups purely matched with tissue types [26]. For samples in a studied population, DMRs occur because of the hypermethylation or hypomethylation of any combination of samples as compared with the remaining samples. Likewise, DMRs identified in different tissues in this study were not specifically hypermethylated or hypomethylated in any particular tissue; some had mixed methylation statuses. This leads to the possibility of using an unsupervised approach to search for different patterns of tissue-specific methylation simultaneously. In addition, our results provide a more global picture of the variation of methylation across tissues and individuals. Comparative studies have identified many genomic regions with tissue-specific methylation and expression that are conserved across different species, such as for the human genome as compared with the mouse [57] and chimpanzee [58] genome. In this study, we found about 6,000 DMRs with consistent methylation statuses across samples for a given tissue. These highly conserved DMRs are of great interest because they suggest an essential role for DNA methylation in regulating differentiation and development of tissues and may reflect tissue-specific patterns of gene expression levels [59]. Among DMRs identified by the ICDMR, nearly 40% were resided in the gene body and might be irrelevant to gene silencing. The mechanistic or functional investigations of these DMRs should be further studied, especially for the regulated mechanism of gene expression level.

## Supporting Information

Figure S1 **Four different instances of correlation between contiguous probes.** (**A**) Neither continuous nor discrete correlation; (**B**) continuous correlation only; (**C**) discrete correlation only, and (**D**) both continuous and discrete correlation. The plots show the log_2_ ratio of methylation intensities observed from the GEO dataset of CpG island hypermethylation in human astrocytomas (accession number GSE19391). Probes are identified by their probe ID. The ellipses indicate the multivariate analogs of the s.d. for each mixture component, estimated using the R package *mclust*.
(PDF)

Figure S2 **Distribution and threshold of concordance.** The figure depicts distributions of (**A**) concordance and (**B**) significant threshold, for α = 0.05, before and after non-biologically relevant correlation correction. The density of concordance is estimated for each ρ value from combined data from 10 simulation repeats. The boxplots depict the variation of threshold across 10 repeats and among different correlations.
(PDF)

Figure S3 **Mean sensitivity and false positive rate given** ***E*** **= 4.** The figure summarizes mean sensitivity (red solid line, left axis) and false positive rate (blue dash line, right axis) for ICDMR, QDMR, t-test and WRST. The mean difference of methylation intensities between methylated and unmethylated groups is 4 (i.e., *E* = 4). The proportion of probes residing in the DMRs is 0.2. At the indicated MF, mean sensitivity and false positive rate are calculated given the correlation between neighboring probes being 0 (ρ = 0), 0.3 (ρ = 0.3), 0.5 (ρ = 0.5) and 0.7 (ρ = 0.7), respectively. Different MF values are indicated on the x-axis.
(PDF)

Figure S4 **Distribution of concordance for human astrocytomas (GSE19391).** The estimated distributions of concordance scores arising from non-DMRs and DMRs are shown in dark and light gray, respectively. The estimated DMR threshold of 0.786 is indicated by a dashed line.
(PDF)

Figure S5 **Distribution of methylation frequency for 5,208 consistently DMRs.** The figure summarizes distribution of methylation frequency for 5,208 consistently DMRs in human astrocytomas. Methylation frequencies of 0.15 and 0.85 are indicated by red dashed lines.
(PDF)

Figure S6 **Distribution of concordance in human tissues.** The estimated distributions of concordance scores arising from non-DMRs and DMRs are shown in dark and light gray, respectively. The estimated DMR threshold of 0.785 is indicated by a dashed line.
(PDF)

Figure S7 **Hierarchical clustering diagram of samples in human astrocytomas.** The diagram shows hierarchical clustering results of samples in human astrocytomas with 176,789 DMRs. The clustering is carried out with pearson distance and complete linkage method.
(PDF)

Figure S8 **Hierarchical clustering diagram of samples in human tissues.** The diagram shows hierarchical clustering results of samples in human tissues with 1,448,166 DMRs. The clustering is carried out with pearson distance and complete linkage method.
(PDF)

Figure S9 **Ankyrin repeat genes.** The graph depicts normalized methylation intensity data for regions near the transcription start site of the eleven ankyrin repeat genes. The tick marks on the genomic coordinate axis indicate genomic positions of the probes designed for the microarray. Blue boxes mark the positions of the genes. The lines represent methylation signals for tumors (red) and normal tissue (blue). The dashed horizontal line indicates methylation intensity at 0. The gray boxes indicate contiguous DMRs identified by ICDMR.
(PDF)

Table S1 **False positive rate and sensitivity given** ***E*** **= 2.**
(PDF)

Table S2 **DAVID annotation in Human astrocytomas.** DAVID Functional Annotation Cluster Analysis of 350 and 304 genes that were close to hyper- and hypomethylation regions in human astrocytomas.
(XLSX)

Table S3 **DAVID annotation in human tissues.** DAVID Functional Annotation Cluster Analysis of 1,051 genes that were close to DMRs found in human tissues.
(XLSX)
