# Supplementary figures and images for: A Novel Method for Identification and Quantification of Consistently Differentially Methylated Regions
Source: PLoS One. 2014 May 12;9(5):e97513. doi: 10.1371/journal.pone.0097513 (PMC4018258; doi:10.1371/journal.pone.0097513)

**A**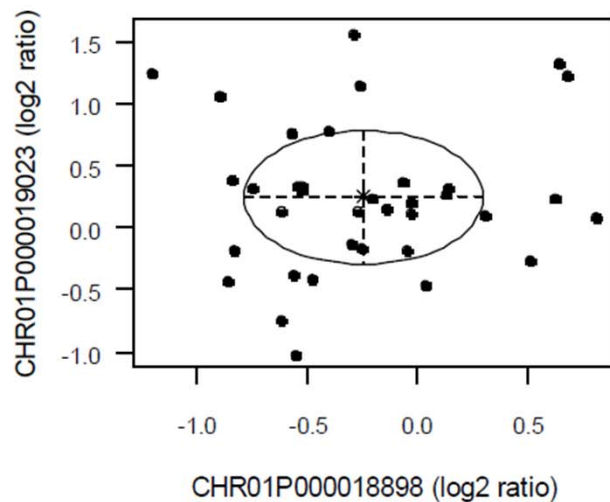**B**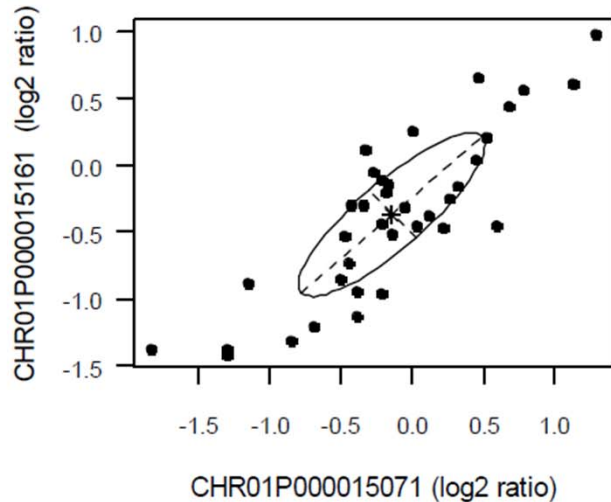**C**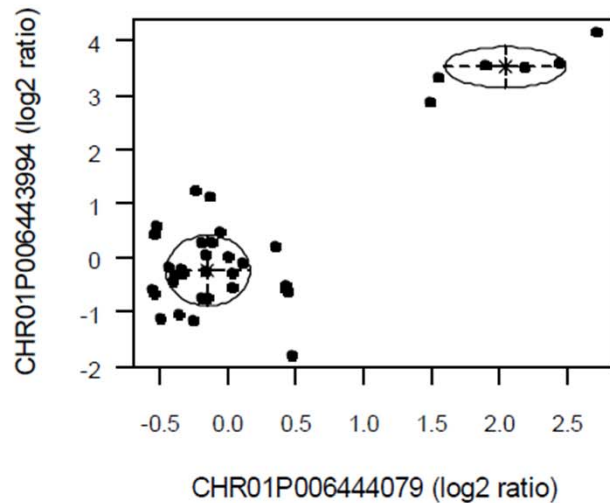**D**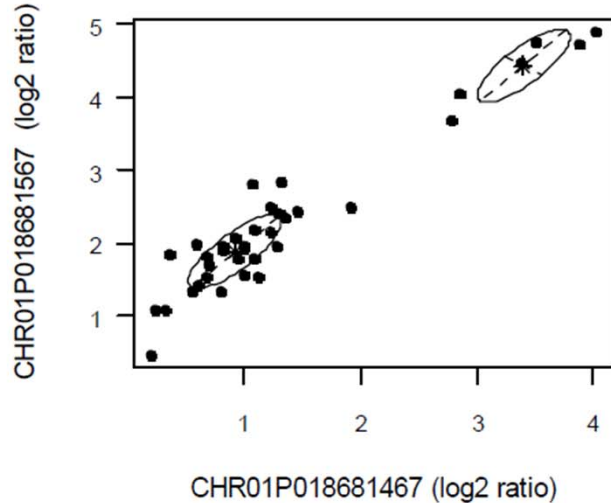

Supplement: Figure S1 — Four different instances of correlation between contiguous probes. (A) Neither continuous nor discrete correlation; (B) continuous correlation only; (C) discrete correlation only, and (D) both continuous and discrete correlation. The plots show the log2 ratio of methylation intensities observed from the GEO dataset of CpG island hypermethylation in human astrocytomas (accession number GSE19391). Probes are identified by their probe ID. The ellipses indicate the multivariate analogs of the s.d. for each mixture component, estimated using the R package mclust. (PDF) [file pone.0097513.s001.pdf]

**A****Before correction**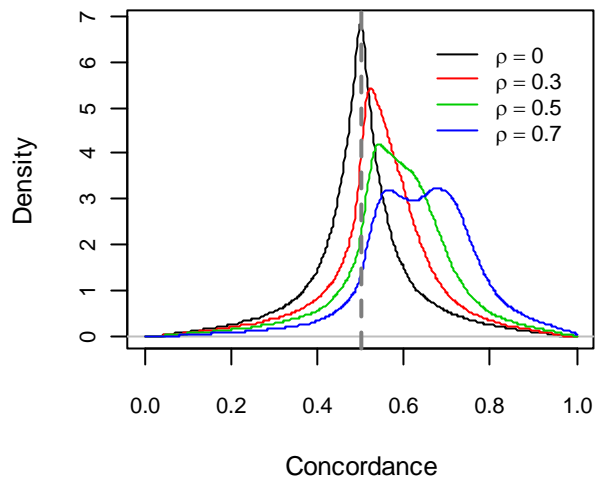**After correction**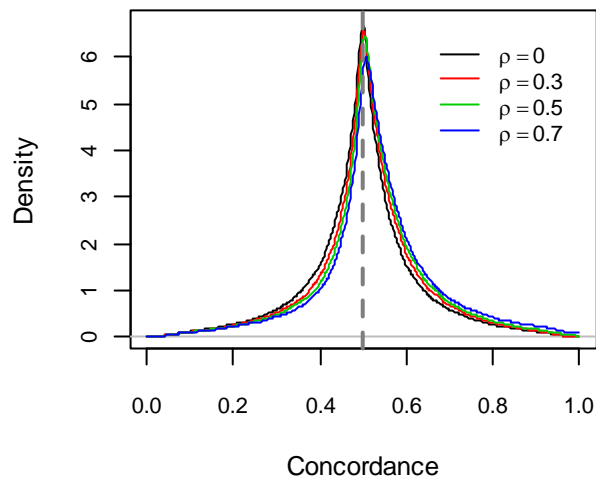**B****Before correction**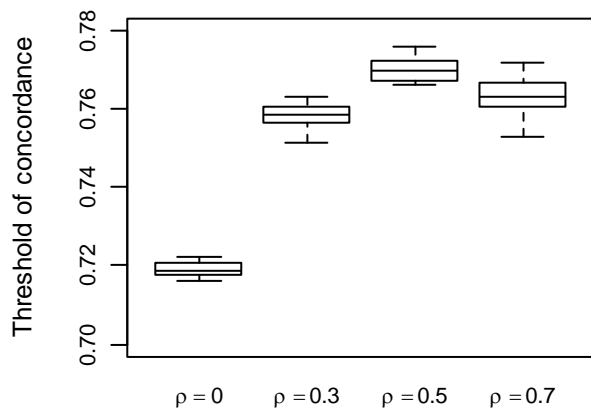**After correction**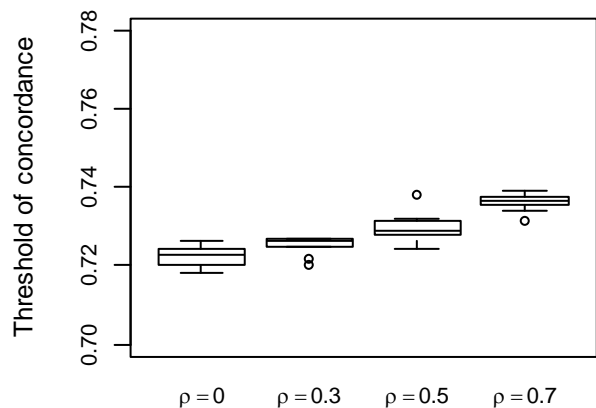

Supplement: Figure S2 — Distribution and threshold of concordance. The figure depicts distributions of (A) concordance and (B) significant threshold, for α = 0.05, before and after non-biologically relevant correlation correction. The density of concordance is estimated for each ρ value from combined data from 10 simulation repeats. The boxplots depict the variation of threshold across 10 repeats and among different correlations. (PDF) [file pone.0097513.s002.pdf]

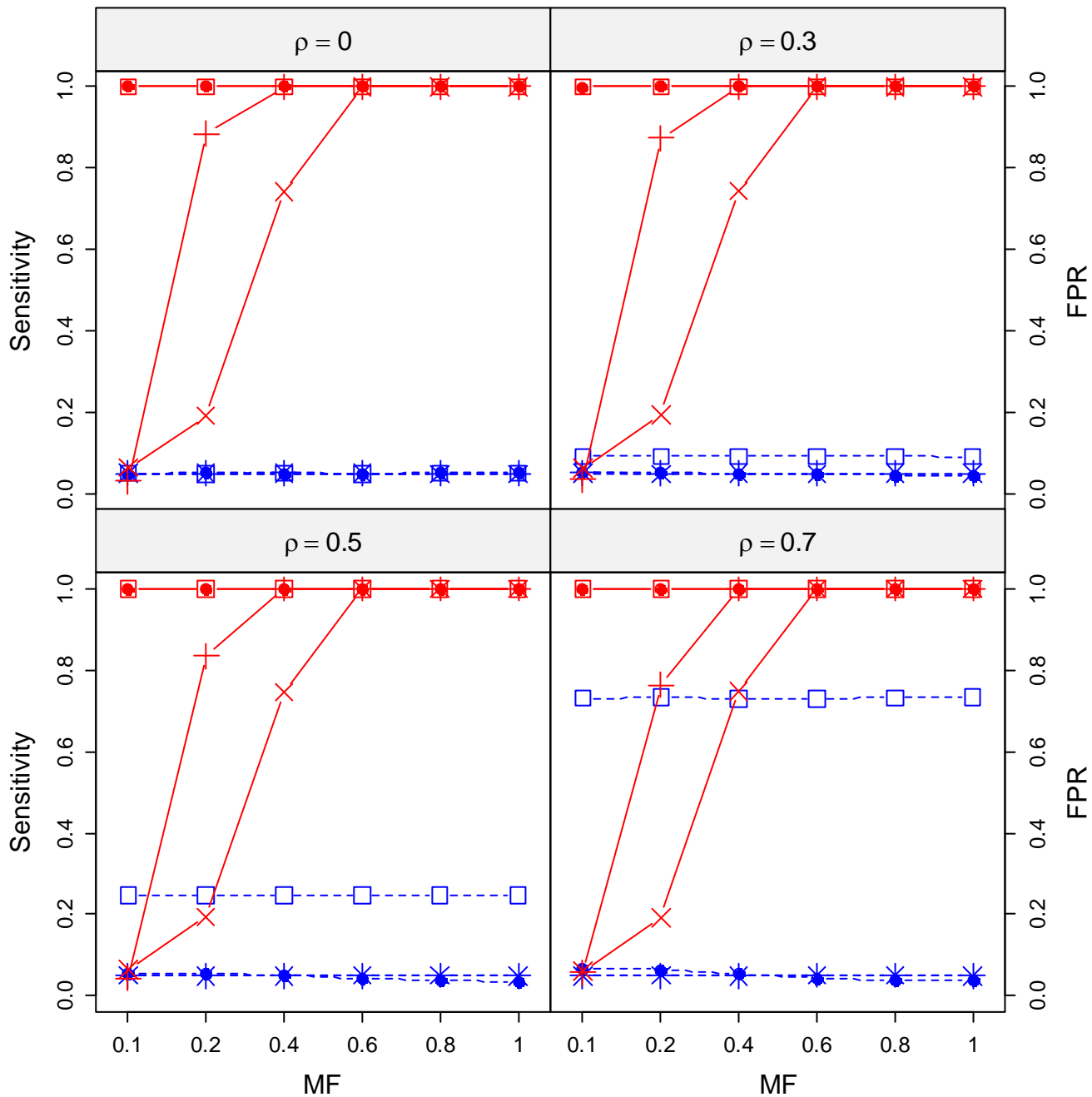

Sensitivity: —●— ICDMR —□— QDMR —+— t-test —x— WRST

FPR: -●- ICDMR -□- QDMR -+- t-test -x- WRST

Supplement: Figure S3 — Mean sensitivity and false positive rate given E = 4. The figure summarizes mean sensitivity (red solid line, left axis) and false positive rate (blue dash line, right axis) for ICDMR, QDMR, t-test and WRST. The mean difference of methylation intensities between methylated and unmethylated groups is 4 (i.e., E = 4). The proportion of probes residing in the DMRs is 0.2. At the indicated MF, mean sensitivity and false positive rate are calculated given the correlation between neighboring probes being 0 (ρ = 0), 0.3 (ρ = 0.3), 0.5 (ρ = 0.5) and 0.7 (ρ = 0.7), respectively. Different MF values are indicated on the x-axis. (PDF) [file pone.0097513.s003.pdf]

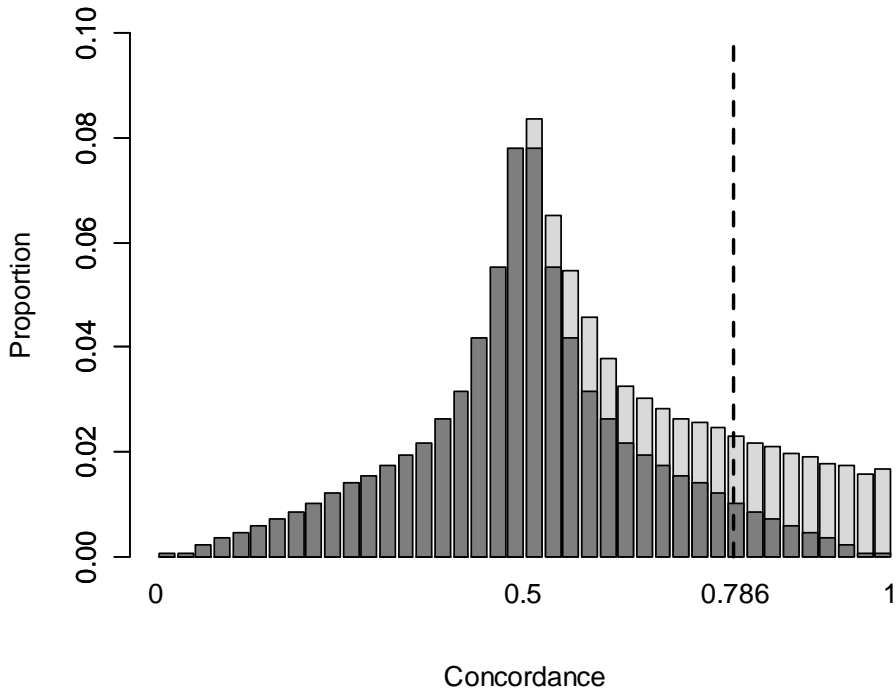

Supplement: Figure S4 — Distribution of concordance for human astrocytomas (GSE19391). The estimated distributions of concordance scores arising from non-DMRs and DMRs are shown in dark and light gray, respectively. The estimated DMR threshold of 0.786 is indicated by a dashed line. (PDF) [file pone.0097513.s004.pdf]

Frequency per 5,208 consistently DMRs

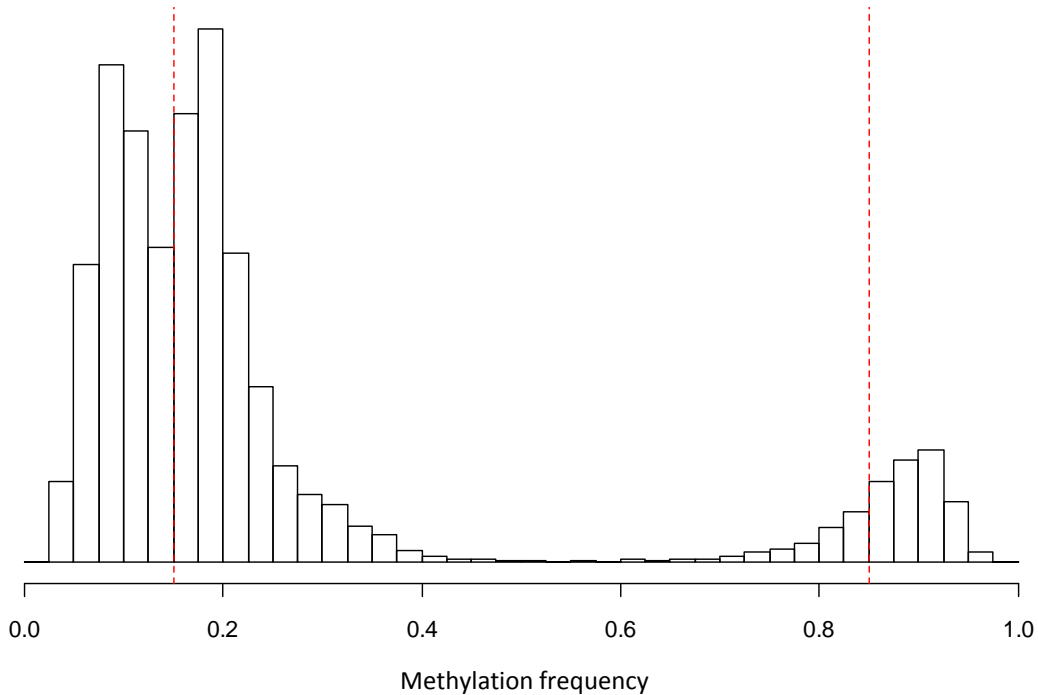

Supplement: Figure S5 — Distribution of methylation frequency for 5,208 consistently DMRs. The figure summarizes distribution of methylation frequency for 5,208 consistently DMRs in human astrocytomas. Methylation frequencies of 0.15 and 0.85 are indicated by red dashed lines. (PDF) [file pone.0097513.s005.pdf]

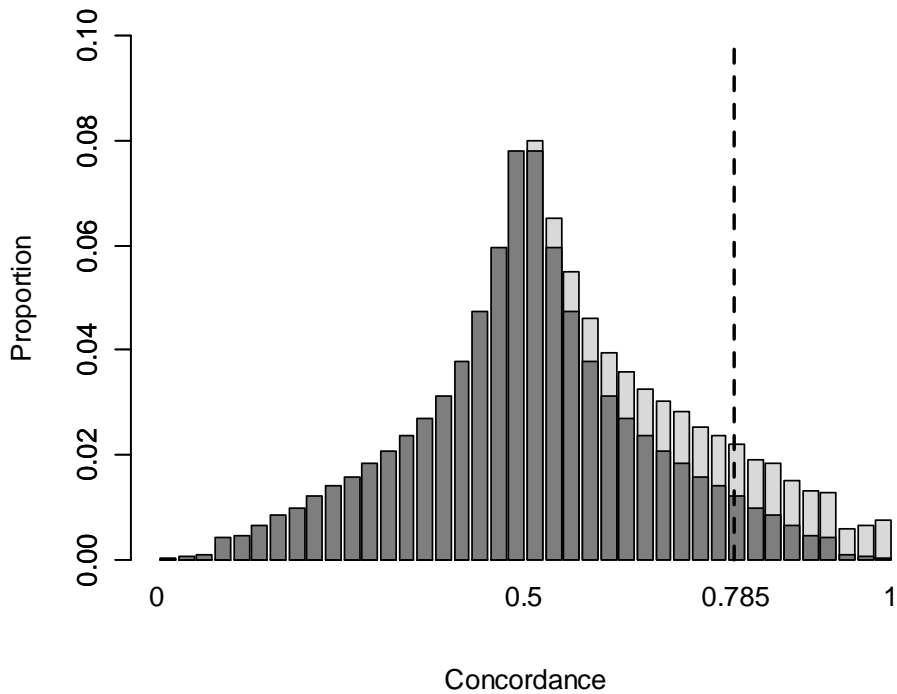

Supplement: Figure S6 — Distribution of concordance in human tissues. The estimated distributions of concordance scores arising from non-DMRs and DMRs are shown in dark and light gray, respectively. The estimated DMR threshold of 0.785 is indicated by a dashed line. (PDF) [file pone.0097513.s006.pdf]

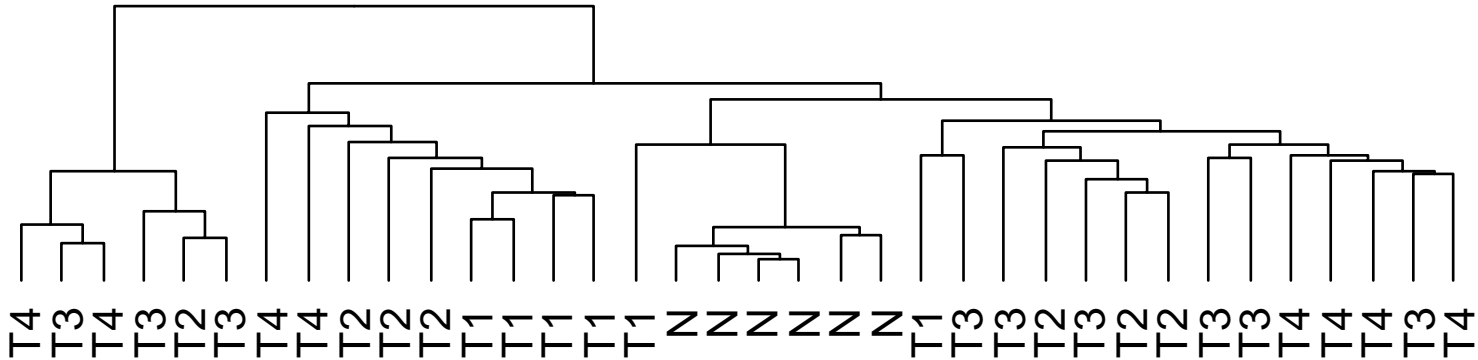

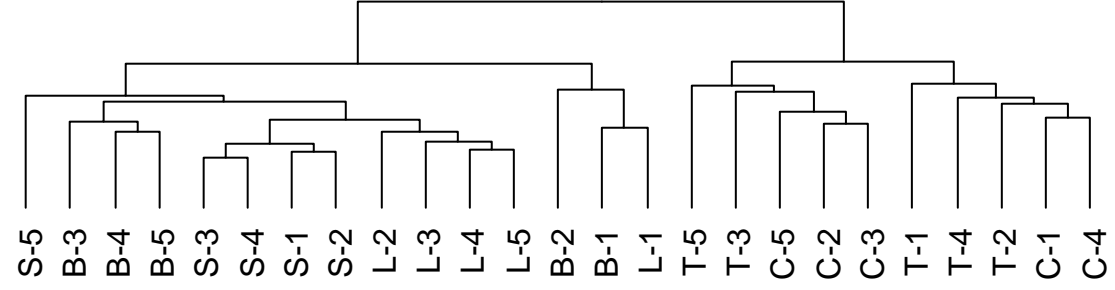

Supplement: Figure S7 — Hierarchical clustering diagram of samples in human astrocytomas. The diagram shows hierarchical clustering results of samples in human astrocytomas with 176,789 DMRs. The clustering is carried out with pearson distance and complete linkage method. (PDF) [file pone.0097513.s007.pdf]

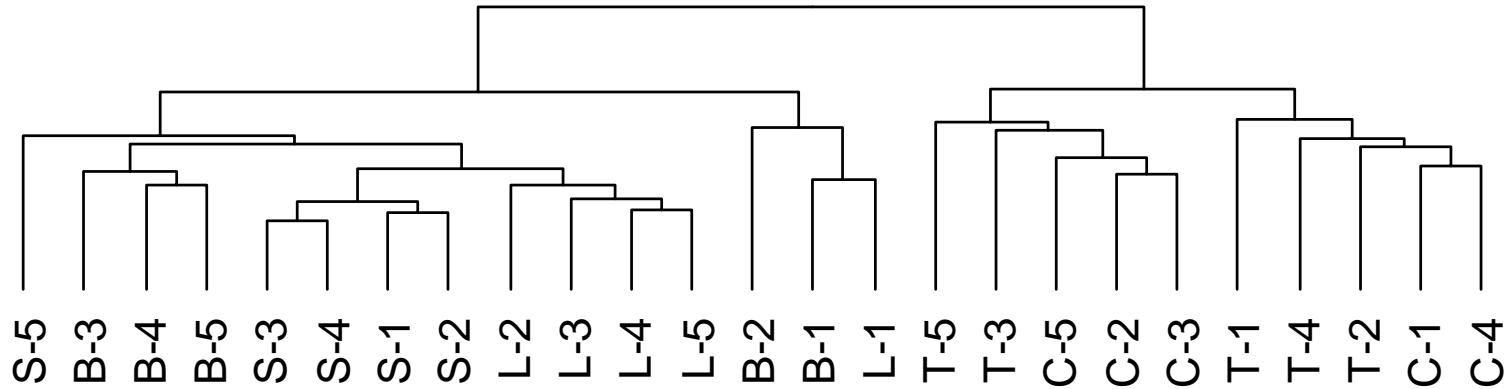

Supplement: Figure S8 — Hierarchical clustering diagram of samples in human tissues. The diagram shows hierarchical clustering results of samples in human tissues with 1,448,166 DMRs. The clustering is carried out with pearson distance and complete linkage method. (PDF) [file pone.0097513.s008.pdf]

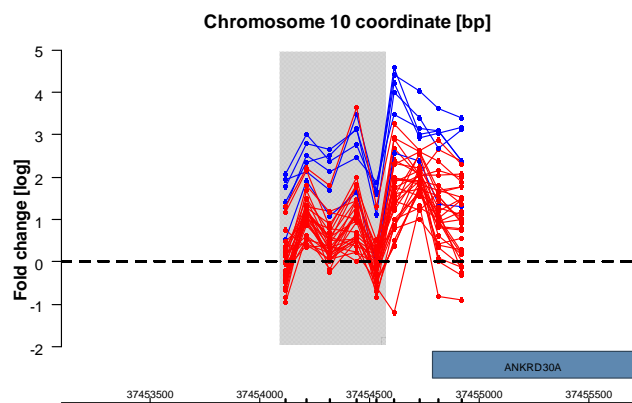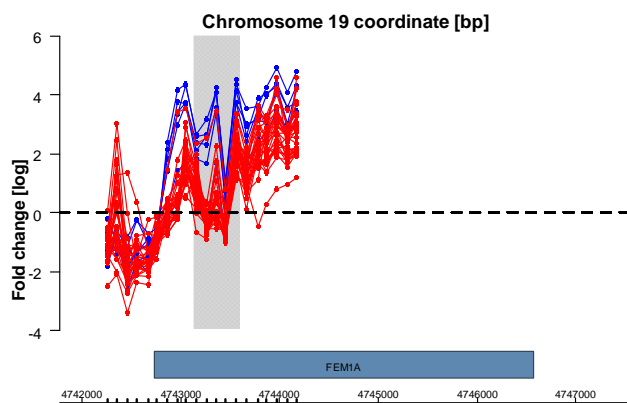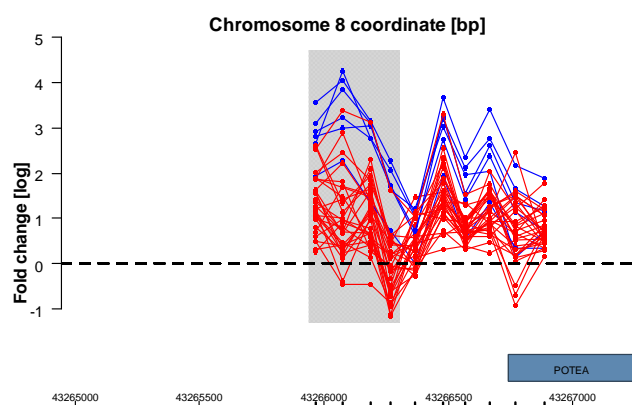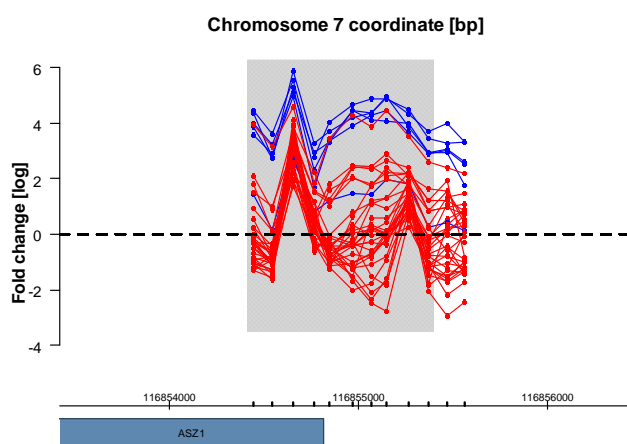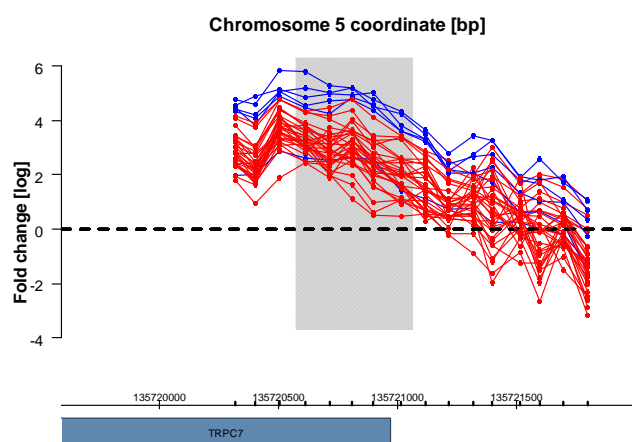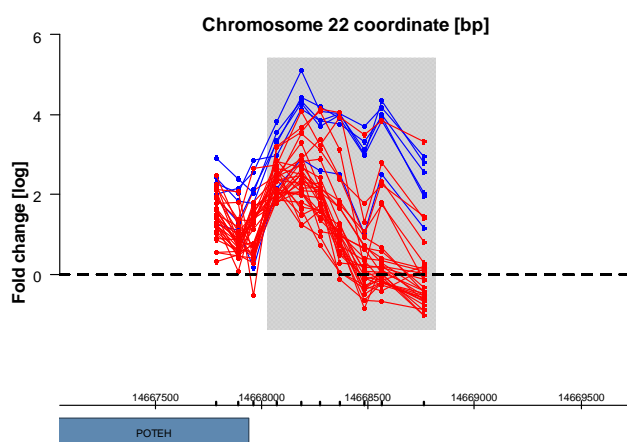

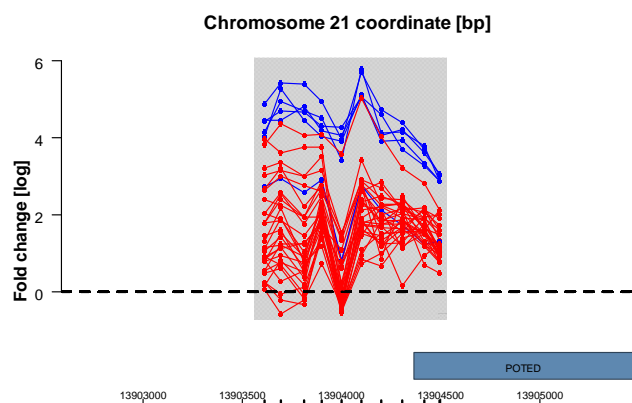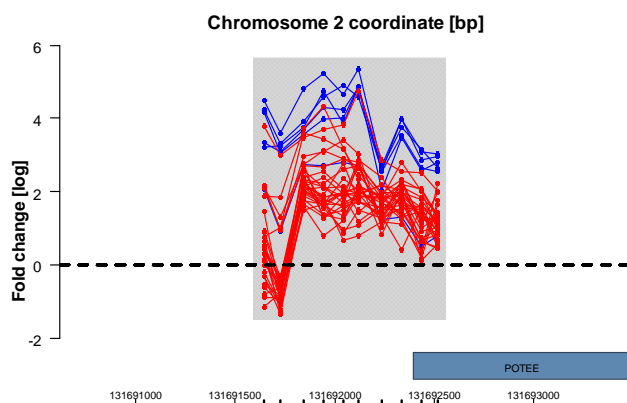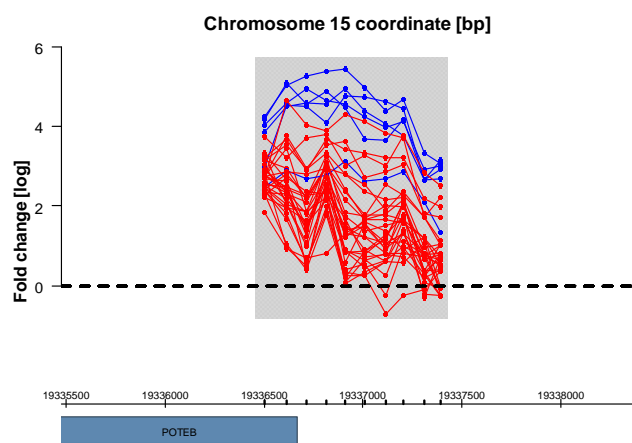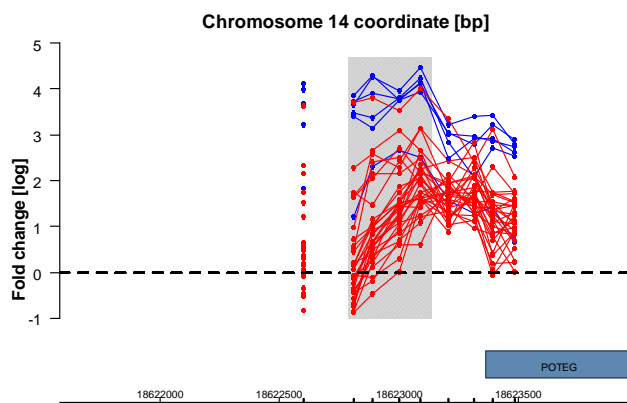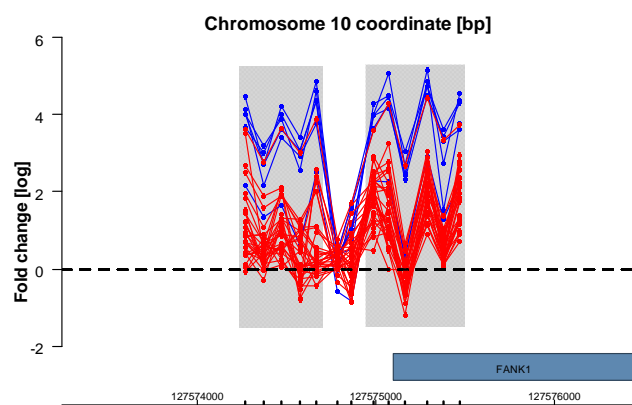

Supplement: Figure S9 — Ankyrin repeat genes. The graph depicts normalized methylation intensity data for regions near the transcription start site of the eleven ankyrin repeat genes. The tick marks on the genomic coordinate axis indicate genomic positions of the probes designed for the microarray. Blue boxes mark the positions of the genes. The lines represent methylation signals for tumors (red) and normal tissue (blue). The dashed horizontal line indicates methylation intensity at 0. The gray boxes indicate contiguous DMRs identified by ICDMR. (PDF) [file pone.0097513.s009.pdf]
