# Supplementary material for: A Novel Method for Identification and Quantification of Consistently Differentially Methylated Regions
Source: PLoS One. 2014 May 12;9(5):e97513. doi: 10.1371/journal.pone.0097513 (PMC4018258; doi:10.1371/journal.pone.0097513)
Supplement: Table S1 — False positive rate and sensitivity given E = 2. (PDF) [file pone.0097513.s010.pdf]

Method	ρ	False positive rate MF 0.1	0.2	0.4	0.6	0.8	1	Sensitivity MF 0.1	0.2	0.4	0.6	0.8	1
**ICDMR**	**0**	0.050	0.050	0.051	0.050	0.049	0.048	0.997	0.999	0.999	0.999	0.999	0.999
	**0.3**	0.052	0.051	0.052	0.052	0.053	0.051	0.994	0.999	0.999	0.999	0.999	0.998
	**0.5**	0.057	0.056	0.054	0.056	0.055	0.052	0.984	0.998	0.998	0.999	0.999	0.996
	**0.7**	0.067	0.067	0.064	0.067	0.063	0.061	0.903	0.990	0.993	0.992	0.991	0.956
**QDMR**	**0**	0.049	0.049	0.049	0.049	0.049	0.049	0.999	1.000	1.000	1.000	1.000	1.000
	**0.3**	0.091	0.091	0.092	0.091	0.090	0.090	0.999	1.000	1.000	1.000	1.000	1.000
	**0.5**	0.248	0.247	0.246	0.248	0.245	0.246	0.999	1.000	1.000	1.000	1.000	1.000
	**0.7**	0.731	0.732	0.731	0.731	0.732	0.730	1.000	1.000	1.000	1.000	1.000	1.000
**t-test**	**0**	0.050	0.049	0.049	0.049	0.049	0.049	0.079	0.612	0.999	1.000	1.000	1.000
	**0.3**	0.049	0.049	0.049	0.050	0.049	0.049	0.082	0.591	0.999	1.000	1.000	1.000
	**0.5**	0.049	0.049	0.050	0.049	0.050	0.050	0.083	0.552	0.999	1.000	1.000	1.000
	**0.7**	0.049	0.050	0.049	0.049	0.049	0.049	0.083	0.470	0.996	1.000	1.000	1.000
**WRST**	**0**	0.049	0.049	0.049	0.049	0.049	0.049	0.061	0.194	0.743	0.999	1.000	1.000
	**0.3**	0.049	0.049	0.049	0.049	0.049	0.049	0.062	0.192	0.748	0.999	1.000	1.000
	**0.5**	0.049	0.049	0.049	0.050	0.049	0.049	0.061	0.192	0.745	0.999	1.000	1.000
	**0.7**	0.049	0.049	0.048	0.049	0.048	0.049	0.061	0.189	0.743	0.999	1.000	1.000
